# Prognostic Analysis of Gastric Signet Ring Cell Carcinoma and Hepatoid Adenocarcinoma of the Stomach: A Propensity Score-Matched Study

**DOI:** 10.3389/fonc.2021.716962

**Published:** 2021-08-11

**Authors:** Yu Yang, Yuxuan Li, Xiaohui Du

**Affiliations:** Department of General Surgery, First Medical Center of Chinese People’s Liberation Army (PLA), General Hospital, Beijing, China

**Keywords:** hepatoid adenocarcinoma of the stomach, signet ring cell carcinoma, overall survival, propensity score matching, prognosis

## Abstract

**Background:**

Hepatoid adenocarcinoma of the stomach (HAS) is a rare type of primary gastric cancer, and most previous studies have reported that HAS has a poor prognosis due to its aggressive biological behavior. The aim of this study was to compare the prognosis of HAS to that of gastric signet ring cell carcinoma (SRC).

**Methods:**

This was a single-center, retrospective, observational cohort study (January 2010 to January 2016) of gastric cancer patients with pathological HAS and SRC. Overall survival was compared between HAS and SRC patients. We used univariate Cox regression, multivariate Cox regression, propensity score matching (PSM), inverse probability of treatment weighting, standardized mortality ratio weighting, standardized mortality ratio weighting, and overlap weighting to perform a prognostic analysis.

**Results:**

A total of 725 (672 SRC and 53 HAS) patients were included. After nearest-neighbor 1:4 PSM, 200 SRC patients and 50 HAS patients were matched. Only in univariate Cox regression analysis with the cohort before PSM did HAS show a significantly worse prognosis than SRC [hazard ratio (HR), 1.66; 95% confidence interval (CI), 1.02–2.69, *p* = 0.040]. However, in the analysis of multivariate Cox regression with the cohort before PSM and series analysis based on the propensity score, all of the results indicated that there was no statistically significant difference in overall survival between HAS and SRC (all *p* > 0.05). Furthermore, in the subgroup of proximal location (*p* = 0.027), T stage 4a & 4b (*p* = 0.001), N stage 3a & 3b (*p* = 0.022), with cancer nodules (*p* = 0.026), serum CEA higher than the normal value (*p* = 0.038), and serum CA199 higher than the normal value (*p* = 0.023), the prognosis of HAS was significantly worse than that of SRC.

**Conclusion:**

Based on our study, there was no statistically significant difference in overall survival between HAS and gastric SRC patients. However, in patients with an advanced tumor stage, HAS may have a worse overall survival than SRC.

## Introduction

Hepatoid adenocarcinoma of the stomach (HAS) is a rare type of primary gastric cancer (GC), and most previous studies have reported that the incidence of HAS is less than 1% of all GC (1, 2). According to the World Health Organization (WHO) gastrointestinal tumor sample classification, hepatoid adenocarcinoma (HAC) is defined as adenocarcinoma of extrahepatic origin with morphological features of liver cell differentiation, composed of large polygonal eosinophilic hepatocytes such as neoplastic cells (3). The etiology of HAS is not clear, and some studies suggest that the occurrence of HAS may be related to the common embryonic origin of the stomach and liver from the foregut (4). HAS is considered to have a poor prognosis due to its aggressive biological behavior (5, 6). However, the prognosis of HAS remains controversial; for example, in the study of Zhou et al., there was no significant difference in the prognosis between HAS and non-HAS GC (7).

According to the WHO Classification of Tumors of the Digestive System, the main categories of gastric adenocarcinoma are tubular and papillary adenocarcinoma (T&PAC), mucinous adenocarcinoma (MAC), signet ring cell carcinoma (SRC), mixed carcinomas, and rare histological variants (8). The incidence of gastric SRC is 15.9%–17% of all GCs (9); moreover, the prognosis of SRC is considered worse than that of other types of GC, especially among patients with advanced cancer stages (10, 11). Therefore, the proportion of SRC in the non-HAS GC population will directly affect the prognosis of non-HAS and thus affect the comparison of the prognosis of HAS and non-HAS GC.

The objective of this study was to compare the prognosis of HAS to that of gastric SRC, explore whether HAS really does have a worse prognosis than SRC, and confirm whether HAS is a subtype of GC with a poor prognosis.

## Materials and Methods

### Study Design and Patient Selection

A single-center retrospective cohort study was conducted utilizing the database of the First Medical Center of Chinese PLA General Hospital from January 1, 2010, to January 1, 2016. A total of 3,095 patients with GC were admitted. The inclusion criteria were SRC or HAS patients according to the final pathology report after radical surgery. The exclusion criteria included the following: patients younger than 18 years or older than 80 years at the time of diagnosis; lack of a pathological diagnosis; the pathological diagnosis was tubular adenocarcinoma, papillary adenocarcinoma, mucinous adenocarcinoma, or another rare type of GC; the tumor tissue contains both SRC and HAS components; with distant metastasis; with palliative surgery; history of prior or concurrent other malignancies; incomplete clinical data; and missing follow-up. The database included information on demographics, clinical and pathological characteristics, and follow-up visits. The follow-up ended on March 1, 2019, and the data were obtained by reviewing medical records and telephone follow-up. Our primary outcome was overall survival (OS), defined as the time from surgery to death from cancer or any other cause. This study was approved by the ethics committee of the Chinese PLA General Hospital and was conducted in accordance with the recommendations of the institutional review board, which waived the requirement for informed consent due to its retrospective nature.

### Diagnosis of Patients

The diagnosis of all patients was confirmed by postoperative pathological diagnosis. SRC was defined as a tumor that only had a signet ring cell carcinoma component or was only mixed with a tubular adenocarcinoma component and/or a papillary adenocarcinoma component. HAS was defined as a tumor that only had a hepatoid adenocarcinoma component or was only mixed with a tubular adenocarcinoma component and/or a papillary adenocarcinoma component.

### Statistical Analysis

To minimize the potential bias of basic clinical characteristics, multivariate Cox regression with propensity score matching (PSM) (12) was used to compare the prognosis between HAS and SRC. A 1:4 nearest-neighbor matching algorithm was applied using a caliper width of 0.2. Fifteen independent variables thought to be confounders were selected to generate the propensity score, and these variables are marked in **Table 1**. A standardized mean difference (SMD) was used to examine the degree of PSM, and a threshold of less than 0.1 was considered acceptable. Survival curves were plotted by Kaplan–Meier and log-rank analyses.

**Table 1:** Baseline characteristics of participants.

Characteristics	Unmatched patients			PSM patients		
	SRC	HAS	SMD	SRC	HAS	SMD
	672	53		200	50	
	No. (%)	No. (%)		No. (%)	No. (%)	
Gender (female)	152 (22.6)	12 (22.6)	0.001	42 (21.0)	12 (24.0)	0.072
Age ≥ 60 years old (yes)	310 (46.1)	31 (58.5)	0.249	115 (57.5)	28 (56.0)	0.030
Neoadjuvant chemotherapy (no)	622 (92.6)	48 (90.6)	0.072	184 (92.0)	46 (92.0)	<0.001
BMI ≥ 24 (no)	369 (54.9)	28 (52.8)	0.042	112 (56.0)	25 (50.0)	0.120
Location			0.179			0.050
Proximal	196 (29.2)	15 (28.3)		52 (26.0)	14 (28.0)	
Middle	176 (26.2)	18 (34.0)		68 (34.0)	17 (34.0)	
Distal	300 (44.6)	20 (37.7)		80 (40.0)	19 (38.0)	
Tumor size ≥ 4 cm (no)	314 (46.7)	23 (43.4)	0.067	92 (46.0)	22 (44.0)	0.040
T stage			0.496			0.028
T1	140 (20.8)	5 (9.4)		20 (10.0)	5 (10.0)	
T2	134 (19.9)	10 (18.9)		38 (19.0)	10 (20.0)	
T3	144 (21.4)	22 (41.5)		78 (39.0)	19 (38.0)	
T4	254 (37.8)	16 (30.2)		64 (32.0)	16 (32.0)	
N stage			0.280			0.078
N0	300 (44.6)	17 (32.1)		70 (35.0)	16 (32.0)	
N1	90 (13.4)	9 (17.0)		32 (16.0)	8 (16.0)	
N2	115 (17.1)	13 (24.5)		48 (24.0)	12 (24.0)	
N3	167 (24.9)	14 (26.4)		50 (25.0)	14 (28.0)	
Lymph nodes examined ≥16 (yes)	532 (79.2)	44 (83.0)	0.098	158 (79.0)	41 (82.0)	0.076
Perineural invasion (no)	548 (81.5)	37 (69.8)	0.276	142 (71.0)	36 (72.0)	0.022
Vascular invasion (no)	505 (75.1)	36 (67.9)	0.161	141 (70.5)	34 (68.0)	0.054
Cancer nodules (no)	619 (92.1)	46 (86.8)	0.174	176 (88.0)	44 (88.0)	<0.001
CEA > 5.0 μg/L (no)	96 (14.3)	9 (17.0)	0.074	27 (13.5)	8 (16.0)	0.071
CA199 > 37.0 U/ml (no)	81 (12.1)	5 (9.4)	0.085	18 (9.0)	5 (10.0)	0.034
CA724 > 10.0 U/ml (no)	80 (11.9)	5 (9.4)	0.080	19 (9.5)	5 (10.0)	0.017

BMI, Body mass index; CEA, Carcinoembryonic antigen; CA, Carbohydrate antigen; HAS, Hepatoid adenocarcinoma of the stomach; PSM, Propensity score matching; SMD, Standardized mean difference; SRC, Signet ring cell carcinoma.

To more reliably compare the differences in overall survival between HAS and SRC, the following survival analysis method and weighting method were performed (1): univariate survival analysis was performed using Cox univariate regression analysis before PSM (2); multivariate Cox regression analysis was performed with adjustments for all covariates shown in **Table 1** before PSM; (3) multivariate Cox regression analysis was conducted with the same strata and covariates after matching according to the propensity score; (4) multivariate Cox regression analysis was conducted with the same strata and covariates and inverse probability of treatment weighting (IPTW) according to the propensity score (13); (5) multivariate Cox regression analysis was conducted with the same strata and covariates and overlap weighting (OW) according to the propensity score (14).

Subgroup analysis was performed with univariate Cox regression analysis after PSM to explore the consistency of the prognostic differences between HAS and SRC in the different subgroups.

All statistical analyses were performed using R-4.0 software (http://www.r-project.org), and *p* < 0.05 (two-sided) was considered statistically significant.

## Results

### Participants

**Figure 1:**
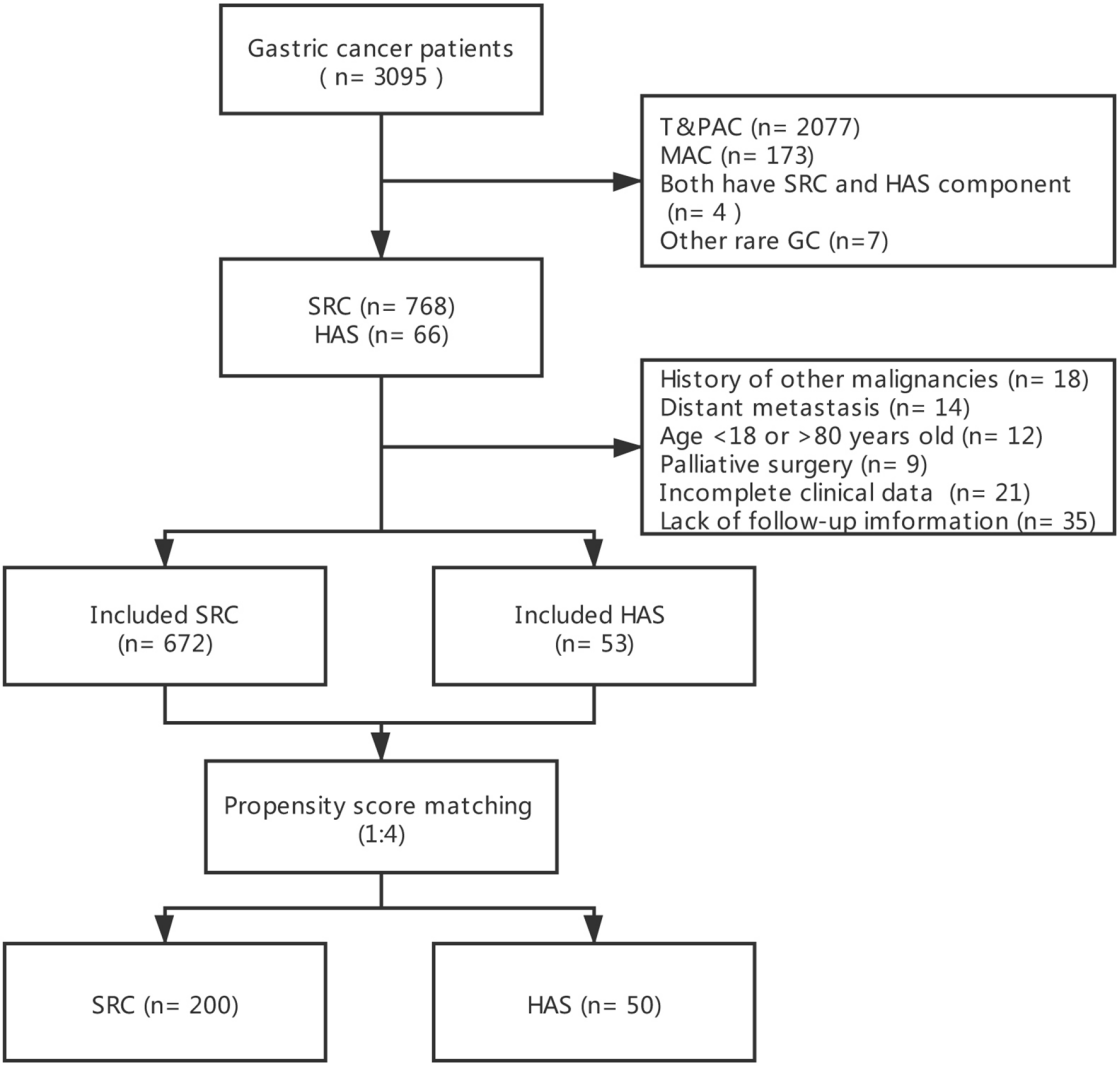
Flow chart depicting the selection of the study population.

Between January 1, 2010, and January 1, 2016, there were 3,095 GC registrations in our medical center. After screening by the inclusion and exclusion criteria, 672 SRC patients and 53 HAS patients remained. After 1:4 PSM, 200 SRC patients and 50 HAS patients were matched. The flow chart depicting the selection of the study population is presented in **Figure 1**.

### Baseline Characteristics

In the crude cohort before PSM, the two groups (HAS and SRC) were consistent in 8 of a total of 15 variables, but there was no consistency in the distribution of age (SMD = 0.249), tumor location (SMD = 0.179), T stage (SMD = 0.496), N stage (SMD = 0.280), perineural invasion (SMD = 0.276), vascular invasion (SMD = 0.161), or cancer nodules (SMD = 0.174). In the matched cohort after PSM, except for body mass index (SMD = 0.120), the other 14 variables were consistent between the two groups (**Table 1**).

### Outcome Analysis

**Figure 2:**
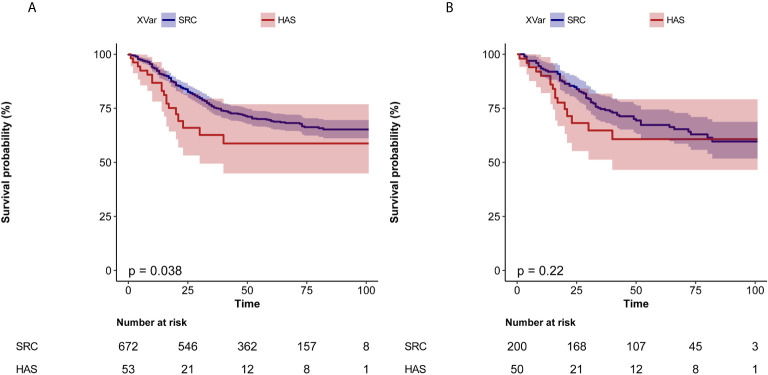
Kaplan–Meier curves for overall survival according to lymph node ratio. **(A)** Before PSM. **(B)** After PSM.

In the crude cohort, the median follow-up was 52 [interquartile range (IQR), 33–74.0] months in the SRC group and 19 (IQR, 16–47) months in the HAS group. The crude 1-year survival was 92.3% [95% confidence interval (CI), 90.3%–94.4%] *vs.* 86.8% (95% CI, 78.1%–96.4%), and the 3-year survival was 75.3% (95% CI, 72.0%–78.7%) *vs.* 62.7% (95% CI, 49.4%–79.5%) in the SRC *vs.* HAS group, respectively. Comparing OS, the HAS group had a worse prognosis. In univariate Cox regression analysis, the hazard ratio (HR) was 1.66 (95% CI, 1.02–2.69, *p* = 0.040) (**Table 2**). Kaplan–Meier curves showed the same outcome (log-rank test: *p* = 0.038) (**Figure 2A**). However, in multivariate Cox regression analysis, OS was not significantly different between the two groups, and the HR was 1.63 (95% CI, 0.99–2.70, *p* = 0.056) (**Table 2**).

**Table 2:** Different analysis methods compare the prognostic differences between HAS and SRC patients in overall survival (HAS *vs.* SRC).

Analysis	HR (95% CI)	*p*-value
Unmatched univariate analysis	1.66 (1.02, 2.69)	0.040
Multivariate adjusted	1.63 (0.99, 2.70)	0.056
Propensity score matched	1.35 (0.96, 1.91)	0.087
Weighted IPTW	1.22 (0.73, 2.05)	0.446
Weighted OW	1.31 (0.65, 2.61)	0.448

IPTW, Inverse probability of treatment weighting; OW, Overlap weighting.

In the matched cohort, the median follow-up was 52 (IQR, 23–73) months in the SRC group and 20 (IQR, 16–48) months in the HAS group. The 1-year survival was 92.4% (95% CI, 88.8%–96.2%) *vs.* 90.0% (95% CI, 82.1%–98.7%), and the 3-year survival was 74.7% (95% CI, 68.8%–81.0%) *vs.* 64.8% (95% CI, 51.3%–81.8%) in the SRC *vs.* HAS group. Multivariate Cox regression analysis after PSM showed that the difference in prognosis between the two groups was not statistically significant (**Table 2**). Kaplan–Meier curves showed the same outcome (log-rank test: *p* = 0.220) (**Figure 2B**).

### Sensitivity Analysis

**Figure 3:**
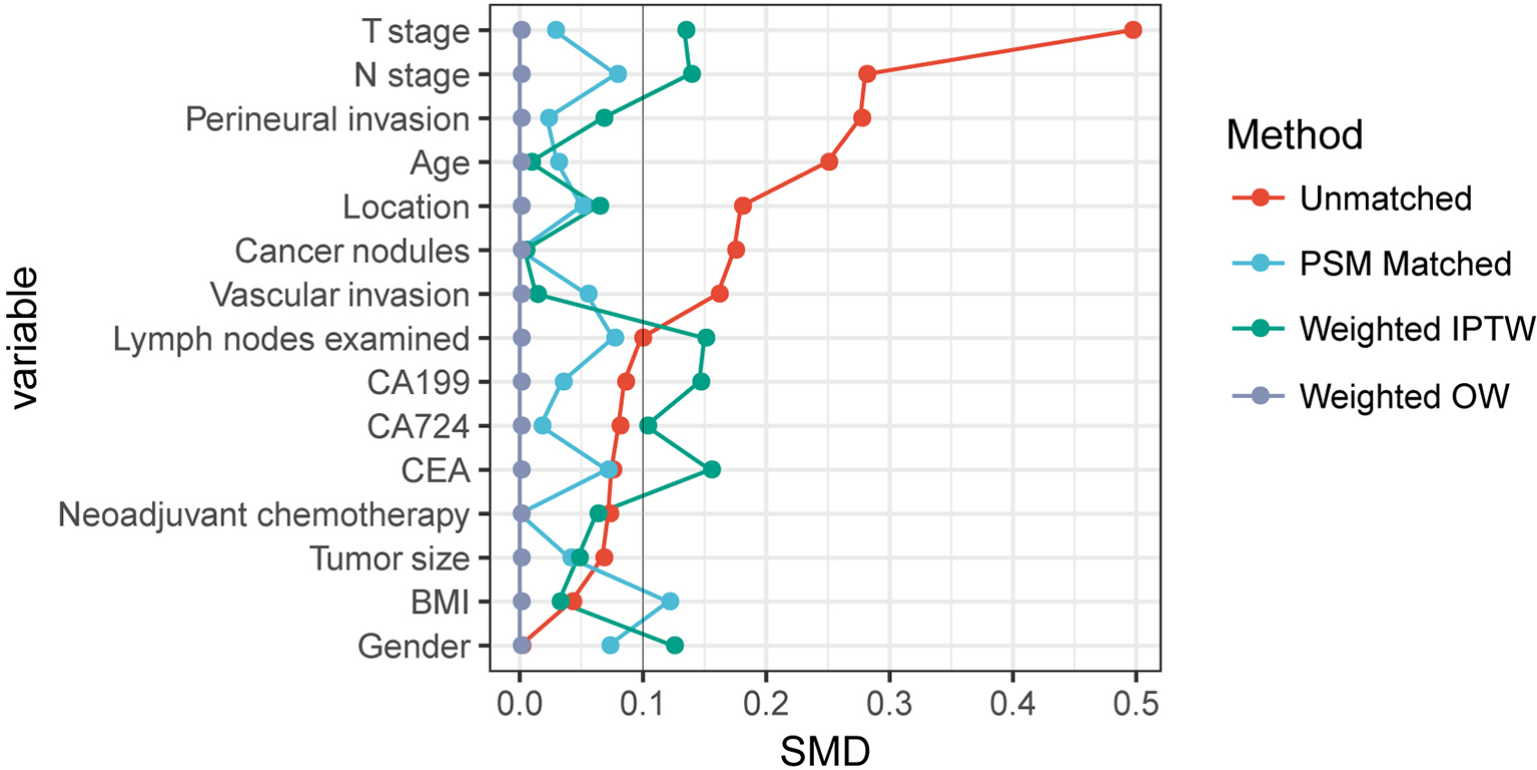
Comparability of baseline characteristics based on standardized mean difference in different survival analysis method.

To further verify the stability of the results, IPTW and OW weighted adjusted multivariate Cox regression analysis according to the propensity score was performed. The baseline characteristics of the two groups were better balanced in these analyses (**Figure 3**). Although all of the results showed that HAS had a worse prognosis than SRC (all HR > 1), the difference was not statistically significant (all *p* > 0.05) (**Table 2**).

### Subgroup Analysis

In the subgroup analysis stratified by the 15 variables, in most subgroups, the prognosis of HAS and SRC was not significantly different. However, in the subgroup of proximal location (*p* = 0.027), T stage 4a & 4b (*p* = 0.001), N stage 3a & 3b (*p* = 0.022), with cancer nodules (*p* = 0.026), serum CEA higher than the normal value (*p* = 0.038), and serum CA199 higher than the normal value (*p* = 0.023), the prognosis of HAS was significantly worse than that of SRC (**Table 3**).

**Table 3:** Subgroup analysis with univariate Cox regression analysis of overall survival between HAS and SRC after PSM.

Subgroup	SRC			HAS			*p*-value
	*n* total	*n* event (%)	HR (95% CI)	*n* total	*n* event (%)	HR (95% CI)	
Gender							
Male	158	54 (34.2)	1 (Ref)	38	12 (31.6)	1.43 (0.76–2.69)	0.263
Female	42	14 (33.3)	1 (Ref)	12	4 (33.3)	1.3 (0.43–3.99)	0.643
Age ≥ 60 years old							
No	85	29 (34.1)	1 (Ref)	22	5 (22.7)	0.99 (0.38–2.57)	0.979
Yes	115	39 (33.9)	1 (Ref)	28	11 (39.3)	1.77 (0.9–3.48)	0.098
Neoadjuvant chemotherapy							
Yes	16	6 (37.5)	1 (Ref)	4	1 (25)	0.74 (0.09–6.18)	0.782
No	184	62 (33.7)	1 (Ref)	46	15 (32.6)	1.45 (0.82–2.56)	0.201
BMI ≥ 24							
Yes	88	32 (36.4)	1 (Ref)	25	9 (36)	1.49 (0.71–3.14)	0.297
No	112	36 (32.1)	1 (Ref)	25	7 (28)	1.26 (0.56–2.85)	0.580
Location							
Proximal	52	14 (26.9)	1 (Ref)	14	6 (42.9)	3.05 (1.13–8.2)	0.027
Middle	68	21 (30.9)	1 (Ref)	17	5 (29.4)	1.56 (0.58–4.18)	0.381
Distal	80	33 (41.2)	1 (Ref)	19	5 (26.3)	0.7 (0.27–1.8)	0.456
Tumor size ≥ 4 cm							
Yes	108	42 (38.9)	1 (Ref)	28	9 (32.1)	1.14 (0.55–2.35)	0.723
No	92	26 (28.3)	1 (Ref)	22	7 (31.8)	1.9 (0.81–4.43)	0.137
T stage							
T 1a & 1b	20	1 (5)	1 (Ref)	5	0 (0)	0 (0–Inf)	0.999
T 2	38	15 (39.5)	1 (Ref)	10	1 (10)	0.41 (0.05–3.15)	0.393
T 3	78	32 (41)	1 (Ref)	19	4 (21.1)	0.7 (0.25–2)	0.511
T 4a & 4b	64	20 (31.2)	1 (Ref)	16	11 (68.8)	3.69 (1.75–7.79)	0.001
N stage							
N 0	70	8 (11.4)	1 (Ref)	16	1 (6.2)	0.6 (0.07–4.83)	0.633
N 1	32	11 (34.4)	1 (Ref)	8	1 (12.5)	0.86 (0.11–6.97)	0.887
N 2	48	19 (39.6)	1 (Ref)	12	5 (41.7)	1.66 (0.61–4.48)	0.318
N 3a &3b	50	30 (60)	1 (Ref)	14	9 (64.3)	2.54 (1.14–5.65)	0.022
Lymph nodes examined ≥16							
Yes	42	15 (35.7)	1 (Ref)	9	1 (11.1)	0.36 (0.05–2.75)	0.325
No	158	53 (33.5)	1 (Ref)	41	15 (36.6)	1.73 (0.97–3.09)	0.062
Perineural invasion							
Yes	58	29 (50)	1 (Ref)	14	4 (28.6)	0.78 (0.27–2.22)	0.639
No	142	39 (27.5)	1 (Ref)	36	12 (33.3)	1.91 (0.99–3.67)	0.052
Vascular invasion							
Yes	59	23 (39)	1 (Ref)	16	8 (50)	2.1 (0.92–4.78)	0.078
No	141	45 (31.9)	1 (Ref)	34	8 (23.5)	1.06 (0.5–2.25)	0.888
Cancer nodules							
Yes	24	12 (50)	1 (Ref)	6	5 (83.3)	3.52 (1.16–10.72)	0.026
No	176	56 (31.8)	1 (Ref)	44	11 (25)	1.17 (0.61–2.24)	0.636
CEA > 5.0 μg/L							
No	173	58 (33.5)	1 (Ref)	42	11 (26.2)	1.1 (0.58–2.11)	0.766
Yes	27	10 (37)	1 (Ref)	8	5 (62.5)	3.61 (1.07–12.16)	0.038
CA199 > 37.0 U/ml							
No	182	62 (34.1)	1 (Ref)	45	12 (26.7)	1.14 (0.61–2.12)	0.681
Yes	18	6 (33.3)	1 (Ref)	5	4 (80)	4.69 (1.24–17.78)	0.023
CA724 > 10.0 U/ml							
No	181	59 (32.6)	1 (Ref)	45	14 (31.1)	1.57 (0.87–2.82)	0.134
Yes	19	9 (47.4)	1 (Ref)	5	2 (40)	0.71 (0.15–3.28)	0.659

AFP, Alpha fetoprotein; BMI, Body mass index; CA Carbohydrate antigen; CEA, Carcinoembryonic antigen; HAS, Hepatoid adenocarcinoma of the stomach; HR, Hazard ratio; SRC, Signet ring cell carcinoma.

## Discussion

HAS is a rare neoplasm, and the annual incidence of HAS is approximately 0.58–0.83 cases per million people (6, 15). Previous studies were mainly case reports or case series from a single medical center and mainly came from Asian regions (1, 2, 16). In these previous studies, HAS patients often were reported to have a worse prognosis than non-HAS patients (17, 18). In this study, a relatively large number of HAS patients were included. Although the prognosis of HAS was significantly worse than that of SRC in the survival analysis without adjusting for confounders, the prognosis of HAS and SRC did not show a significant difference in multivariate regression analysis. In addition, in other analyses based on propensity scores, the results were consistent, and the prognosis of HAS was not statistically worse than that of SRC. Therefore, based on these results, we inferred that there was no difference in overall survival between HAS and SRC.

In the subgroup analyses, the results were very interesting. Although in most subgroups HAS did not show a difference in prognosis from SRC, in some subgroups of indicators suggesting an advanced stage of the tumor (T stage 4a & 4b, N stage 3a & 3b, with cancer nodules, serum CEA higher than the normal value, and serum CA199 higher than the normal value), HAS had a worse overall survival than SRC. At present, the controversy about the prognosis of SRC lies in previous studies showing that the prognosis of SRC in early-stage patients may be better than that of non-SRC (19, 20), while the prognosis of SRC in advanced-stage patients is worse (10, 11). The reason for the worse prognosis of overall SRC patients has been suggested to be caused by a greater proportion of patients in an advanced stage (21). However, in our study, it seems that in patients with an advanced tumor stage, the overall survival of HAS was worse than that of SRC.

In a subgroup analysis based on tumor location, in the proximal GC group, the comparison of the prognosis between HAS and SRC was significantly different. Analyzing the reasons for this result, it is unavoidable that the reliability of the result is limited due to the scant sample size, but at the same time, we should also consider the impact of the differences in biological characteristics between proximal GC and distal GC. Previous studies have shown that proximal GC and distal GC have differences in their expression of some oncogenes and antioncogenes, such as HER2 (22), Smad4 (23), p53 (24), and p16 (23). This reminds us that in follow-up studies, these factors should be included in the analysis of prognosis.

The lymph nodes examined were related to the prognosis of GC (25), but the optimal number of lymph nodes examined remains controversial (26). The AJCC 8th GC staging system recommends that at least 16 lymph nodes should be examined (27). Obviously, not all patients can have a sufficient number of lymph nodes detected due to the surgical methods applied and for other reasons. In our study, after adjusting for this important confounder, in the subgroup analysis stratified by the lymph nodes examined <16 or ≥16, no prognostic difference was observed between HAS and SRC. This result verified the reliability of our speculation that the two groups had no significant difference in overall survival.

It should be pointed out that after PSM, the covariate BMI did not match well (SMD > 0.1). However, in the subsequent subgroup analysis stratified by BMI, the results were consistent in both layers, and the prognoses of HAS and SRC were not significantly different. This result indicated that the poor matching of BMI did not significantly affect the reliability of the result.

There were several limitations of this study. First, although we adjusted for as many possible confounders as we could and performed a propensity score-matched cohort to balance these confounders between groups, some residual confounders may still exist. Second, the sample size might be small for robust statistical analyses, but HAS is a rare subtype of GC. Most previous studies were case reports or case series, and as far as we know, the largest sample size in a single center report is only 75 cases (7); therefore, further multicenter studies of HAS are necessary. Third, this study only focused on overall survival, not investigating other indicators, such as complications, and the lack of data on local recurrence precluded us from assessing the difference in disease recurrence and disease-free survival. Fourth, some inevitable issues, such as information biases, might exist owing to its retrospective design.

In conclusion, this is the first study to our knowledge to investigate the difference in prognosis between HAS and gastric SRC. Our data suggested that there was no statistically significant difference in the overall survival between patients with HAS and gastric SRC. However, in patients with advanced tumor stages, HAS may have a worse overall survival than SRC.

## Data Availability Statement

The raw data supporting the conclusions of this article will be made available by the authors, without undue reservation.

## Ethics Statement

This study was approved by the ethics committee of the Chinese PLA General Hospital and was conducted in accordance with the recommendations of the institutional review board, which waived the requirement for informed consent due to its retrospective nature.

## Author Contributions

The authors that contributed to the study conception and design were YY, YL, and XD. Data acquisition and interpretation were performed by YY and YL. The first draft of the manuscript was written by YY. All authors contributed to the article and approved the submitted version.

## Conflict of Interest

The authors declare that the research was conducted in the absence of any commercial or financial relationships that could be construed as a potential conflict of interest.

## Publisher’s Note

All claims expressed in this article are solely those of the authors and do not necessarily represent those of their affiliated organizations, or those of the publisher, the editors and the reviewers. Any product that may be evaluated in this article, or claim that may be made by its manufacturer, is not guaranteed or endorsed by the publisher.
